# Prevalence of lower extremity venous duplication

**DOI:** 10.4103/0971-3026.69367

**Published:** 2010-08

**Authors:** William L Simpson, David M Krakowsi

**Affiliations:** Department of Radiology, Mount Sinai Medical Center, Box 1234, New York, NY 10029 USA

**Keywords:** Clot, deep venous thrombosis, Doppler ultrasound, duplication

## Abstract

**Purpose:**

This retrospective study was performed to determine the prevalence of lower extremity venous duplication using duplex ultrasound in the patient population of a large urban medical center.

**Material and Methods:**

The reports of all lower extremity venous ultrasound examinations performed at our institution between January 1, 2002 and December 31, 2002 were reviewed. Ultrasound examinations that were performed for purposes other than the detection of lower extremity deep vein thrombosis were excluded. The prevalence of duplication and its specific location were recorded. In addition, the prevalence of thrombus and its specific location were also recorded.

**Results:**

A total of 3118 exams were performed in 2664 patients. Of the 2664 patients, 2311 had only one examination performed during the study period; 353 patients had more than one examination performed. We found that 10.1% of patients (270/2664) had at least one venous segment duplicated and 5.4% of patients (143/2664) had a thrombus in at least one venous segment. There was a statistically significant difference in the prevalence of both duplication and thrombus with a change in venous segment. Only 0.4% of patients (11/2664) had thrombus within a duplicated segment. Of those who had more than one examination performed, 15.3% (54/353) had the same venous segment(s) seen on one examination but not another.

**Conclusion:**

Lower extremity venous duplication is a frequent anatomic variant that is seen in 10.1% of patients, but it may not be as common as is generally believed. It can result in a false negative result for deep vein thrombosis.

## Introduction

Deep venous thrombosis (DVT), along with pulmonary embolism (PE), constitutes a disease process known as venous thromboembolic disease (VTD). Most PEs are thought to originate in deep vein thrombi in the lower extremities. The thrombus forms in the calf veins, propagates into the femoral-popliteal system, and subsequently embolizes to the lungs. Approximately 300,000 new cases of VTD are reported each year, with 100,000 cases of PE.[1] Approximately 30% of new cases of VTD die within 30 days and 20% die a sudden death due to PE.[2] Treatment of DVT before embolization can prevent PE.

The diagnosis of DVT based on clinical signs and symptoms is notoriously fallible.[3,4] Duplex USG with gray-scale, color Doppler, and compression USG is the noninvasive test of choice for the diagnosis of DVT. Duplex USG has been shown to have 100% sensitivity and 99% specificity for the detection of DVT in the femoral-popliteal system.[5] A limitation of USG is its operator dependency. In addition, duplication in the femoral-popliteal venous system can lead to false negative studies for the diagnosis of DVT, and can therefore be considered a specific limitation of lower extremity Doppler USG. Prior studies based on venography indicate that the rate of duplication in the femoral-popliteal system ranges from 5 to 46%,[6,7] whereas USG studies show a 9 to 25%[8,9] prevalence of duplication.

The purpose of this study was to determine the prevalence of duplicated lower extremity veins using duplex USG in the patient population of a large urban academic medical center.

## Materials and Methods

The reports of all venous Doppler studies performed in the radiology department at our medical center between January 1, 2002 and December 31, 2002 were reviewed. The images were reviewed if there was any ambiguity in the report. Studies that were performed for purposes other than detection of lower extremity DVT were excluded. The study was approved by the institutional investigational review board, with a waiver of informed consent due to the retrospective nature of the study.

During the study period, 3582 venous Doppler studies were performed. Of these, 464 were excluded because they were not performed for the detection of lower extremity DVT; specifically, 350 were performed for the detection of upper extremity DVT, 75 were performed for detection or characterization of a groin pseudoaneurysm, 17 were nonvascular studies incorrectly scheduled as vascular ones, 15 were excluded because they were incomplete evaluations of the femoral-popliteal venous system, three were performed for evaluation of the pelvic veins, and two studies were for evaluation of the inferior vena cava; two other studies were excluded for other reasons. Thus, 3118 lower extremity venous Doppler studies met our criteria for inclusion in the study.

All lower extremity venous Doppler studies were performed on an HDI 5000 (Advanced Technology Laboratories, Andover, MA) or a Sequoia 512 (Acuson, Mountain View, CA) sonography unit using 5 to 7 MHz linear or 4 to 6 MHz curved-array transducers. The studies that were performed during normal business hours were carried out by sonography technicians having 1 to 6 years experience in performing venous Doppler studies. Studies that were performed after working hours and on weekends and holidays were done by second- or third-year radiology residents. Each study consisted of gray-scale evaluation of the veins in both the transverse and longitudinal planes to detect intraluminal thrombus, color Doppler with spectral tracing to document the waveform and respiratory variation, and compression USG of the common femoral vein (CFV), femoral vein (FV), and popliteal vein (PopV). The FV was divided into the following three segments: the proximal FV was the most cranial third, near the CFV; the mid femoral vein (mFV) was the middle third of the vein; and the distal FV was the most caudal third, near the adductor canal. Either one lower extremity or both were interrogated based on the clinical indication or clinician preference. The presence of duplication and its specific location were recorded. For the purpose of this study, any number of vein moiety more than one (two, three, or more) was considered as duplication. The presence of thrombus and its specific location were also documented. The studies were interpreted by board-certified attending radiologists with subspecialty training in body imaging and 3 to 30 years post-training experience in performing and interpreting venous Doppler studies.

To determine whether there were associations between two dichotomous variables, such as gender and leg observed or duplication and thrombus, or in any instance where two proportions were compared, the chi square analysis was utilized. To discern whether the distribution of segmental duplication was similar, a weighted least squares method for one population regression analysis of marginal proportions was performed. The goal was to answer the question: Does the prevalence of duplication change with vein segment location? This is a type of repeated-measures analysis for categorical data, because we were considering five vein segments per patient. The same procedure was followed to explore the formation of thrombus. All data were analyzed using SAS system software (SAS Institute Inc, Cary, NC).

## Results

A total of 3118 studies were performed in 2664 patients (1593 women, 1071 men; age range, 2 weeks – 105 years, mean age, 60.5 years). Of the 2664 patients, 2311 patients had only one study performed during the study period, whereas 353 had more than one study performed. Of the 3118 studies, imaging of both femoral-popliteal venous systems was performed in 1692 (54%), whereas 1426 (46%) surveyed only one extremity. Of these unilateral studies, 686 were performed on the left femoral-popliteal venous system and 740 on the right. Thus, imaging was performed for a total of 4810 limbs and 24,050 venous segments.

### Duplications

**Figure 1 (A,B):**
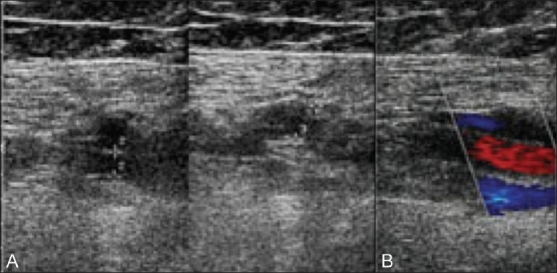
A 77-year-old female with duplication in the left femoral vein. Transverse gray-scale image (A) of the left mid femoral vein (delineated by electronic calipers) demonstrates duplication, with one moiety below the artery and a smaller one above the artery. Both moieties are compressible. Longitudinal color Doppler image demonstrates color flow (coded blue) within both the anterior and posterior moieties (B)

In this study, 1163 patients underwent a unilateral lower extremity venous Doppler study; 596 had a study of the left limb and 567 had a study of the right limb. Of these, 88 had duplication in at least one segment [Table 1]. Of the 1501 patients who had a bilateral lower extremity venous Doppler study, 182 had duplication in at least one segment. Thus, 10.1% (270/2664) of the patients in this study had at least one duplicated segment [Figure 1]. The most commonly duplicated single segment was the mFV [Table 1]. Using the weighted least squares method, we found a statistically significant difference in the prevalence of duplication with a change in vein segment (*P*<0.0001).

**Table 1:** Distribution of lower extremity venous duplication

	CFV	pFV	mFV	dFV	POP	Total
Unilateral						
R or L	0	48	61	38	11	158
Bilateral						
Right	0	22	27	13	1	63
Left	0	50	53	28	2	133
Both	0	44	59	22	5	130
Total	0	164	200	101	19	484

R: right, L: left, CFV: Common femoral vein, pFV: proximal femoral vein, mFV: mid femoral vein, dFV: distal femoral vein; POP - popliteal vein.

### Thrombi

**Figure 2 (A,B):**
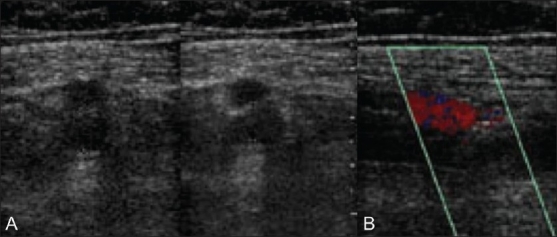
A 66-year-old male with thrombosis of the right femoral vein. Transverse gray-scale image (A) of the proximal right mid femoral vein demonstrates echogenic material within the noncompressible vein (delineated by electronic calipers). Longitudinal color Doppler image (B) demonstrates no color flow within the thrombosed vein

Of the 1163 patients with unilateral lower extremity venous Doppler studies, 122 (10.5%) had a thrombus [Figure 2] in at least one segment [Table 2]. Using the weighted least squares method, we found a statistically significant difference in the prevalence of thrombus with a change in vein segment [*P*<0.001]. Of the 88 patients with unilateral studies who had at least one duplicated segment, 19 had a thrombus in at least one segment (21.6%). There was a statistically significant difference (*P* = 0.0004) when we compared patients with unilateral studies who had a thrombus in a limb with no duplication (9.6%) and those who had a thrombus as well as a duplication (21.6%).

**Table 2:** Distribution of lower extremity venous thrombus

	CFV	pFV	mFV	dFV	POP	Total
Unilateral						
R or L	63	65	65	66	69	328
Bilateral						
Right	24	20	17	21	24	106
Left	42	35	35	33	40	185
Both	29	27	25	31	27	139
Total	158	147	142	151	160	758

R: right, L: left, CFV: Common femoral vein, pFV: proximal femoral vein, mFV: mid femoral vein, dFV: distal femoral vein, POP: popliteal vein

**Figure 3 (A-C):**
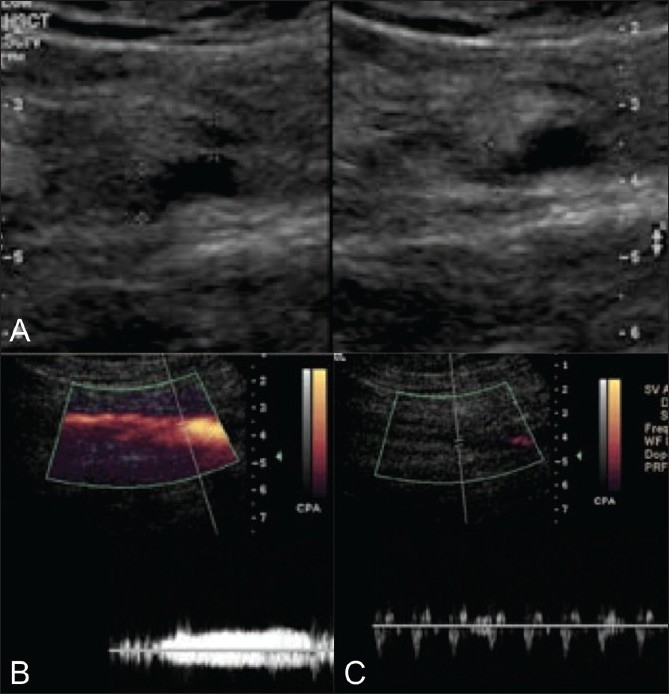
A 34-year-old female with duplication of the right femoral vein and thrombus within one moiety. Transverse gray-scale image (A) of the right mid femoral vein demonstrates duplication; one moiety is compressible and the other is noncompressible and shows internal echogenic material (delineated by electronic calipers on the compression image). Longitudinal power Doppler images demonstrates flow with a venous spectral tracing within the patent moiety (B) and no flow within the thrombosed moiety (C). Spectral tracing shows transmitted pulsation from the adjacent femoral artery

Of the 1501 patients who had bilateral lower extremity venous Doppler studies, 21 (1.4%) had a thrombus in at least one segment. Using the weighted least squares method, we found a statistically significant difference in the prevalence of thrombus with a change in vein segment (*P*<0.001). Of the 184 patients with bilateral studies who had at least one duplicated segment in either limb or both, 21 patients had a thrombus in at least one segment (11.4%) of either or both limbs. There was no statistically significant difference (*P*= 0.85) between patients with bilateral studies who had a thrombus in a limb with no duplication (10.9%) and those who had a thrombus along with a duplication (11.4%). Only 11 patients in the study had a thrombus seen within a duplicated segment [Figure 3]. Thus, the prevalence of thrombus within a duplicated segment was 0.4%.

### Repeat examinations

A total of 353 patients had more than one lower extremity venous Doppler examination performed during the study period. These patients had a total of 807 examinations performed as follows: 277 patients had two studies; 58 patients had three studies; 14 patients had four studies; two patients had five studies; and one patient each had six and seven studies. The repeat examinations were not analyzed as to whether each was a unilateral or bilateral study. The number of patients who had the same duplicated segment(s) seen on one study but not another was 54 (15.3%), with the following distribution: 44 of the 277 patients with two studies; seven of the 58 patients with three studies; one of the 14 patients with four studies; and the two patients with five studies. Interestingly, the patients with six and seven studies each did not have a duplication seen on one study that was also not seen on the other studies, that is, there was 100% agreement on duplicated segments in those with the highest number of repeat exams.

## Discussion

Much of the literature on lower extremity venous duplication is based on venography studies. The prevalence in these studies varies widely, from 5%[8] for the PopV to 46%[7] for the FV. Invasive venography was replaced by duplex USG using gray-scale, compression USG, and color Doppler imaging. USG studies have demonstrated that the prevalence of duplication ranges from 9 to 25%. Many of these US studies were published in the early 1990s using USG technology that cannot be considered state of the art by current standards. Our study, using duplex USG, demonstrated a 10.1% prevalence of duplication within the femoral-popliteal venous system of the lower extremities.

To the best of our knowledge, this is the largest USG study of the lower extremity venous system with over 2600 patients (4810 limbs). The 10.1% prevalence is in line with the 9.1% prevalence reported in the study of 800 patients (1600 limbs) by Kerr *et al*. It is similar to the study of Dona *et al*. which demonstrated a prevalence of 15.7% in a study population of 177 patients (248 limbs). Gordon *et al*. demonstrated a duplication prevalence of 25%; however, that study examined only 58 patients (116 limbs). The larger studies, which are likely more representative of the general population, tend to show lower prevalence rates.

Similar to other studies,[6–9] our study confirmed that the FV is the one that is most commonly duplicated. Moreover, the mid segment is more commonly duplicated than the other segments [Table 1]. Caution should be exercised when considering a potential duplication of the PopV, because a high confluence of the posterior tibial veins can be confused with a true popliteal duplication. There was a statistically significant difference in the prevalence of the location of the duplication when all the different potentially duplicated segments of the femoral-popliteal venous system were compared. In addition, when patients who had more than one USG performed in the study period were analyzed, 15.3% had a duplicated segment that was not seen on at least one previous or subsequent study. This is significant, given that missing duplication on USG is one reason for a false negative result in DVT study. Interestingly, the two patients who had the greatest number of repeat studies, six and seven ultrasound studies each, did not have any duplication missed. The reason for this unexpected discrepancy is not clear.

Thrombus was seen in at least one segment of 5.4% of the patients in the study. Again, there was a statistically significant difference in the prevalence in the location of thrombus when the various potentially thrombosed segments were compared. Thrombus and a duplicated segment were seen in 14.7% (40/272); however, only 0.4% of patients had a thrombus in a duplicated segment.

This study had several limitations. As the study was conducted in an academic medical center, most of the venous Doppler examinations that were done after working hours and on weekends during the study period were performed by second- and third-year radiology residents, whereas during daytime hours the examinations were performed by USG technologists. Thus, the pool of sonographers was diverse in ability. As USG is an operator-dependent modality, some duplicated vein segments may have been missed. In addition, the examinations that comprised our study group were interpreted by seven different board-certified attending radiologists. Some of those radiologists considered a duplicated vein segment to be a normal anatomic variant and did not always include the finding in the report (personal communication). A large patient body habitus or marked edema of a lower extremity can also decrease the sensitivity of the examination for visualization of the veins on gray-scale imaging, particularly the mid and distal segments of the FV. An uncooperative patient can also result in a suboptimal study for visualization of all venous segments. In this study population, 193 examinations (6.2%) were limited in at least one of the aforementioned ways. Therefore, our 10.1% prevalence may be a slight underestimation of the actual prevalence. However, since most of these factors are related to the intrinsic limitations of the imaging modality and would apply to previous studies using USG, we do not feel this significantly decreased the actual prevalence, especially in view of the large sample size in this study.

In conclusion, this is the largest study reported to date of the prevalence of lower extremity venous duplication using duplex USG. The prevalence of duplication of 10.1% is similar to that reported by other large USG-based studies but lower than most venography studies and smaller USG- based studies. The FV is most commonly duplicated, with the mFV being the most frequently duplicated segment. In addition, 15.3% of the studies had a duplicated segment missed on a previous or subsequent examination. Almost 15% of patients had a study showing both thrombus and a duplicated segment in the same limb; however, thrombus within a duplicated segment was extremely rare, occurring in only 0.4% of patients. Duplication of the lower extremity venous system is the anatomic variation which is the most common reason for a false negative DVT study that consequently results in failure to diagnose VTD and PE.
